# Zipf’s Law for Word Frequencies: Word Forms versus Lemmas in Long Texts

**DOI:** 10.1371/journal.pone.0129031

**Published:** 2015-07-09

**Authors:** Álvaro Corral, Gemma Boleda, Ramon Ferrer-i-Cancho

**Affiliations:** 1 Centre de Recerca Matemàtica, Bellaterra, Barcelona, Spain; 2 Departament de Matemàtiques, Universitat Autònoma de Barcelona, Bellaterra, Barcelona, Spain; 3 Department of Translation and Language Sciences, Universitat Pompeu Fabra, Barcelona, Spain; 4 Complexity and Quantitative Linguistics Lab, Departament de Ciències de la Computació, Universitat Politècnica de Catalunya, Barcelona, Spain; University of Gävle, SWEDEN

## Abstract

Zipf’s law is a fundamental paradigm in the statistics of written and spoken natural language as well as in other communication systems. We raise the question of the elementary units for which Zipf’s law should hold in the most natural way, studying its validity for plain word forms and for the corresponding lemma forms. We analyze several long literary texts comprising four languages, with different levels of morphological complexity. In all cases Zipf’s law is fulfilled, in the sense that a power-law distribution of word or lemma frequencies is valid for several orders of magnitude. We investigate the extent to which the word-lemma transformation preserves two parameters of Zipf’s law: the exponent and the low-frequency cut-off. We are not able to demonstrate a strict invariance of the tail, as for a few texts both exponents deviate significantly, but we conclude that the exponents are very similar, despite the remarkable transformation that going from words to lemmas represents, considerably affecting all ranges of frequencies. In contrast, the low-frequency cut-offs are less stable, tending to increase substantially after the transformation.

## Introduction

Zipf’s law for word frequencies is one of the best known statistical regularities of language [1, 2]. In its most popular formulation, the law states that the frequency *n* of the *r*-th most frequent word of a text follows

$$n\left(r\right)\propto \frac{1}{r^{\alpha }},$$ (1)

where *α* is a constant and ∝ the symbol of proportionality. However, Eq (1) is not the only possible approach for modeling word frequencies in texts. One could also look at the number of different words with a given frequency in a text. In that case, the probability *f*(*n*) that a word has frequency *n* is given by

$$f\left(n\right)\propto \frac{1}{n^{\gamma }},$$ (2)

where *γ* is a constant. The real values of *f*(*n*) and *n*(*r*) contain the information about the frequency of the words in a text, but *f*(*n*) does it in a compressed fashion (given only the values of *f*(*n*) such that *f*(*n*) > 0, *n*(*r*) is retrieved for any value of *r*). In the first version of the law, *r*, the so-called rank of a word, acts as the random variable, and in the second version the random variable is the frequency of a word, *n*. In both cases, *α* and *γ* are the exponents, related by [2]

$$\gamma =1+\frac{1}{\alpha }.$$ (3)

Usually, *α* is close to 1 and then *γ* is close to 2.

The relevance of Zipf’s law for human language [3–5], as well as for other species’ communication systems [6–8], has been the topic of a long debate. To some researchers, Zipf’s law for frequencies is an inevitable consequence of the fact that words are made of letters (or phonemes): Zipf’s law is obtained no matter if you are a human or another creature with a capacity to press keys sequentially [3, 4] or to concatenate units to build words in a more abstract sense [6]. This opinion is challenged by empirical values of *α* that are not covered by simple versions of random typing [9], the dependence of *α* upon language complexity during language ontogeny [10], and also by the large differences between the statistics defined on ranks (e.g., the mean rank) and the random typing experiments with parameters for which a good fit was claimed or expected [5].

If the law is not inevitable, understanding the conditions under which it emerges or varies is crucial. Alterations in the shape and parameters of the law have been reported in child language [10, 11], schizophrenic speech [11, 12], aphasia [13, 14], and large multiauthor texts [15–17]. Despite intense research on Zipf’s law in quantitative linguistics and complex systems science, little attention has been paid to the elementary units for which Zipf’s law should hold. Zipf’s law has been investigated in letters [18] and also in blocks of symbols (e.g., words or letters) [19]. Here a very important issue that has not received enough attention since the seminal work of Zipf is investigated in depth: the effect of considering word forms vs. lemmas in the presence, scope and parameters of the law (a lemma is, roughly speaking, the stem form of a word; see below for a more precise definition). Research on this problem is lacking as the overwhelming majority of empirical research has focused on word forms for simplicity (e.g., [10, 16, 17, 20–22, 24]).

Thus, here we address a very relevant research question: does the distribution of word frequencies differ from that of lemmas? This opens two subquestions:

- Does Zipf’s law still hold in lemmas?
- Does the exponent of the law for word forms differ from that of lemmas?

It is remarkable that Zipf himself addressed this problem at a very preliminary level (Fig. 3.5 in Ref. [1]), and it has not been until much more recently that several researchers have revisited it. Baroni compared the distribution of ranks in a lemmatized version of the *British National Corpus* against the non-lemmatized counterpart and concluded, based upon a qualitative analysis, that both show essentially the same pattern [25]. Reference [26] studied one English text (*Ulysses*, by James Joyce) and one Polish text; for the former, the word and lemma rank-frequency relations were practically undistinguishable, but for the Polish text some differences were found: the exponent *α* slightly increased (from 0.99 to 1.03) when going from words to lemmas and a second power-law regime seemed to appear for the highest ranks, with exponent *α* about 1.5. Bentz et al. [27], for a translation of the *Book of Genesis* into English, pointed to a connection between morphology and rank-frequency relations, provided by an increase in the exponent *α* (from 1.22 to 1.29) when the book was lemmatized and Mandelbrot’s generalization of Zipf’s law was used in a maximum likelihood fit of *n*(*r*). Finally, Hatzigeorgiu et al. [28] analyzed the Hellenic National Corpus and found that the exponent *α* decreased when taking the 1000 most frequent units (from *α* = 0.978 for the 1000 most frequent word forms to *α* = 0.870 for the 1000 most frequent lemmas). This decrease is hard to compare with the increases reported in Refs. [26, 27] and the results presented in this article because it is restricted to the most frequent units.

Our study will provide a larger scale analysis, with 10 rather long single-author texts (among them some of the longest novels in the history of literature) in 4 different languages, using state-of-the-art tools in computational linguistics and power-law fitting. The languages we study cover a fair range in the word-lemma ratio, from a morphologically poor language such as English to a highly inflectional language such us Finnish, with Spanish and French being in between. In a previous study with a subset of these texts, some of us investigated the dependence of word and lemma frequency distributions on text length [29], but no direct quantitative comparison was performed between the results for word and lemmas. It will be shown here that the range of validity of Zipf’s law [Eq (2)] decreases when using lemmas; however, we will show that, while the exponents obtained with word forms and lemmas do not follow the same distribution, they maintain a very close and simple relationship, suggesting some robust underlying mechanism.

We will study the robustness of Zipf’s law concerning lemmatization from the perspective of type frequencies instead of ranks. Ranks have the disadvantage of leading to a histogram or spectrum that is monotonically decreasing by definition. This can hide differences between real texts and random typing experiments [30]. The representation in terms of the distribution of frequencies *f*(*n*) has been used successfully to show the robustness of Zipf’s exponents as texts size increases: Although the shape of the distribution apparently changes as text length increases, a simple rescaling allows one to unravel a mold for *f*(*n*) that is practically independent from text length [29]. In this article we investigate the extent to which *f*(*n*) is invariant upon lemmatization. We restrict our analysis to single-author texts, more concretely literary texts. This is because of the alterations in the shape and parameters of the distribution of word frequencies known to appear in large multi-author corpora [15–17].

## Definitions

Let us consider, in general, a sequence composed of symbols that can be repeated. We are studying texts composed by words, but the framework is equally valid for a DNA segment constituted by codons [31], a musical piece consisting of notes [32], etc. Each particular occurrence of a symbol is called a token, whereas the symbol itself is referred to as a type [20]. The total number of tokens gives the sequence length, *L* (the text length in our case), whereas the total number of types is the size of the observed vocabulary, *V*, with *V* ≤ *L*.

In fact, although a sequence may be perfectly defined, its division into symbols is, up to a certain point, arbitrary. For instance, texts can be divided into letters, morphemes, etc., but most studies in quantitative linguistics have considered the basic unit to be the word. This is a linguistic notion that can be operationalized in many languages by delimiting sets of letters separated by spaces or punctuation marks. Nevertheless, the symbols that constitute themselves a sequence can be non-univocally related to some other entities of interest, as it happens with the relationship between a word and its lemma. A lemma is defined as a linguistic form that stands for or represents a whole inflectional morphological paradigm, such as the plural and singular forms of nouns or the different tensed forms of a verb. Lemmas are typically used as headwords in dictionaries. For example, for a word type, *houses*, the corresponding lemma type is *house*. Nevertheless, this correspondence is not always so clear [33], such that lemmatization is by no means a straightforward transformation.

Using different texts, we will check the validity of Zipf’s law for lemmas, and we will compare the statistics of word forms to the statistics of lemmas. To gather statistics for lemmas, we will replace each word in the text by its associated lemma, and will consider the text as composed by lemmas. To see the effect of this transformation, consider for instance the word *houses* in *Ulysses*. The number of tokens for the word type *houses* is 26, because *houses* occurs 26 times in the book. However, the number of tokens for the corresponding lemma, *house*, is 198, because the lemma *house* (in all its nominal and verbal forms, *house, houses, housed*…) occurs 198 times. The relationship between the statistics of words and lemmas, and in particular the question of whether lemmas follow Zipf’s law or not [1], is not a trivial issue [33].

In order to investigate the validity of Zipf’s law in a text we count the frequency *n* of all (word or lemma) types and fit the tail of the distribution of frequencies (starting at some point *n* = *a*) to a power law, i.e.,

$$\begin{array}{c} f\left(n\right)=\frac{C}{n^{\gamma }},\;\;\text{for}\;\;n\geq a, \end{array}$$

with *γ* > 1, *C* the normalization constant, and disregarding values of *n* below *a*. The version of Zipf’s law that we adopt has two parameters: the exponent *γ* and the low-frequency cut-off *a*. We consider that Zipf’s law is valid if a power law holds starting at *a* and reaching at least two decades up to the maximum frequency (the frequency of the most common type). With these assumptions, we are adhering to the view of Zipf’s law as an asymptotic property of a random variable [34, 35].

To fit this definition of the law we use a two-step procedure that first fits the value of *γ* for a fixed *a* and next evaluates the goodness of the power-law fit from *a* onwards; this is repeated for different *a*-values until the most satisfactory fit is found. The resulting exponent is reported as *γ* ± *σ*, where *σ* is the standard deviation of *γ*. Our procedure is similar in spirit to the one by Clauset et al. [23], but it can be shown to have a better performance for continuous random variables [36–38]. Indeed, Clauset et al.’s requirement for power-law acceptance seems to be very strict, having been found to reject the power-law hypothesis even for power-law simulated data [37]. Details of the procedure we use are explained in Ref. [39]; this is basically the adaptation of the method of Ref. [38] to the discrete case. The *Materials and Methods* Section provides a summary.

## Results

We analyze a total of 10 novels comprising four languages: English, Spanish, French, and Finnish, see Table 1. In order to gather enough statistics, we include some of the longest novels ever written, to our knowledge. For the statistical analysis of lemmas, we first perform an automatic process of lemmatization using state of the art computational tools. The steps comprise tokenization, morphological analysis, and morphological disambiguation, in such a way that, at the end, each word token is assigned a lemma. See Materials and Methods for further details.

**Table 1:** Characteristics of the books analyzed. The length of each book *L* is measured in millions of tokens.

Title	Author	Language	Year	*L*
Clarissa^1^	Samuel Richardson	English	1748	0.976
Moby-Dick^2^	Herman Melville	English	1851	0.215
Ulysses	James Joyce	English	1918	0.269
Don Quijote^3^	Miguel de Cervantes	Spanish	1605	0.381
La Regenta	L. Alas “Clarín”	Spanish	1884	0.308
Artamène^4^	Scudéry siblings^9^	French	1649	2.088
Le Vicomte de Bragelonne^5^	A. Dumas (father)	French	1847	0.699
Seitsemän veljestä^6^	Aleksis Kivi	Finnish	1870	0.081
Kevät ja takatalvi^7^	Juhani Aho	Finnish	1906	0.114
Vanhempieni romaani^8^	Arvid Järnefelt	Finnish	1928	0.136

^1^Clarissa: Or the History of a Young Lady.

^2^Moby-Dick; or, The Whale.

^3^El ingenioso hidalgo don Quijote de la Mancha (1605)—The Ingenious Gentleman Don Quixote of La Mancha (title in English); including second part: El ingenioso caballero don Quijote de la Mancha (1615).

^4^Artamène ou le Grand Cyrus—Artamène, or Cyrus the Great.

^5^Le Vicomte de Bragelonne ou Dix ans plus tard—The Vicomte of Bragelonne: Ten Years Later.

^6^Seven Brothers.

^7^Spring and the Untimely Return of Winter.

^8^The Story of my Parents.

^9^Madeleine and Georges de Scudéry.

### Zipf’s law holds for both word forms and lemmas

**Fig 1:**
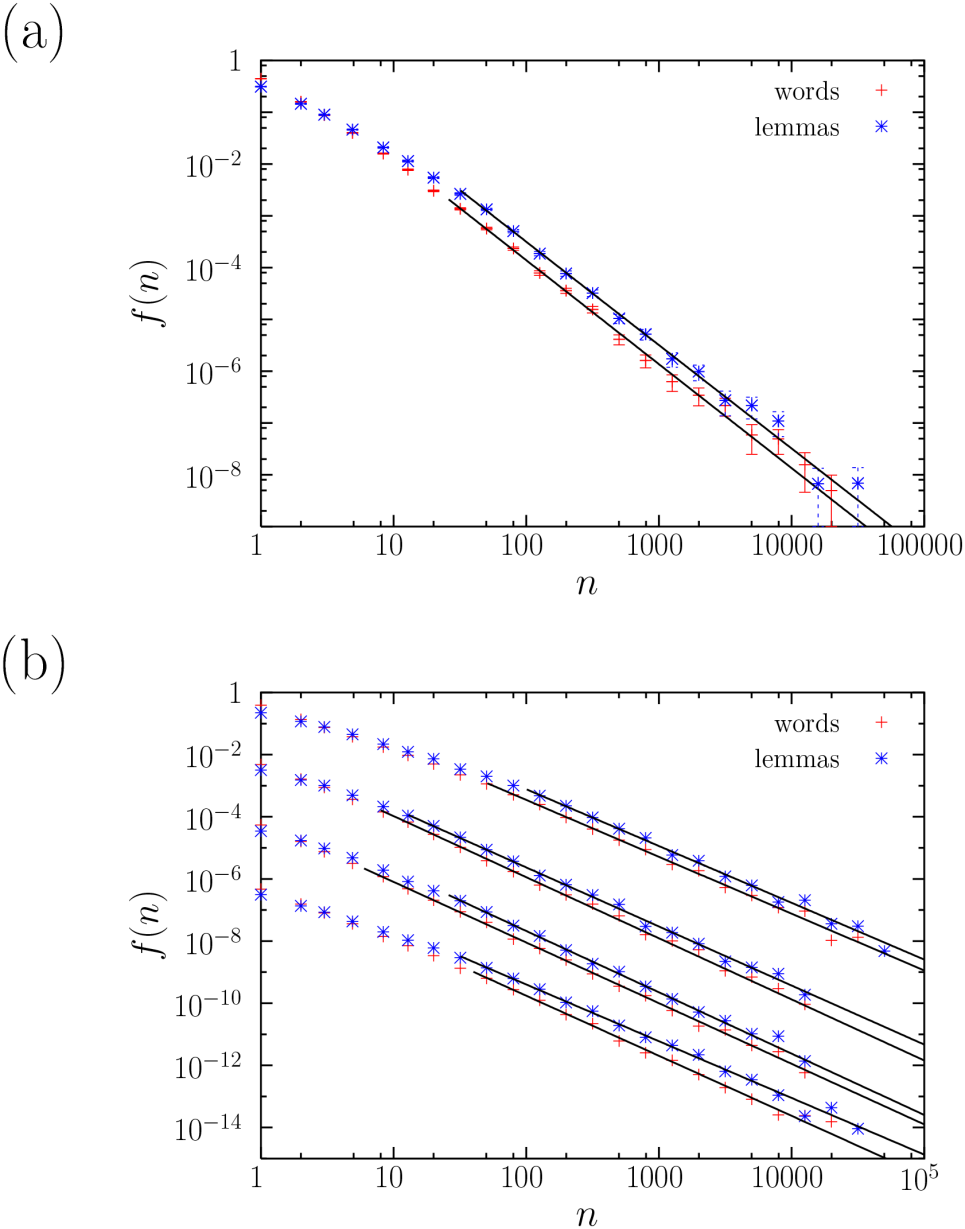
(a) Probability mass functions *f*(*n*) of the absolute frequencies *n* of words and lemmas in *La Regenta*, together with their fits. (b) The same, from top to bottom, for *Clarissa, Moby-Dick, Ulysses* (all three in English), and *Don Quijote* (in Spanish). The distributions are multiplied by factors 1, 10^−2^, 10^−4^ and 10^−6^ for a clearer visualization.

Fig 1(a) compares the results before and after lemmatization for the book *La Regenta* (in Spanish). The frequency distributions *f*(*n*) for words and for lemmas are certainly different, with higher frequencies being less likely for words than for lemmas, an effect that is almost totally compensated by *hapax legomena* (types of frequency equal to one), where words have more weight than lemmas. This is not unexpected, as the lemmatization process leads to less types (lower *V*), which must have higher frequencies, on average (the mean frequency is ⟨*n*⟩ = *L*/*V*). The reduction of vocabulary for lemmas (for a fixed text length) has a similar effect to that of increasing text length; in other words, we are more likely to see the effects of the exhaustion of vocabulary (if this happens) using lemmas rather than words. The difference in the counts of frequencies results in a tendency of *f*(*n*) for lemmas to bend downwards as the frequency decreases towards the smallest values (i.e., the largest ranks) in comparison with the *f*(*n*) of words; this in agreement with Ref. [26]. Besides, one has to take into account that lemmatization errors are more likely for low frequencies, and then the frequency distribution in that domain can be more strongly affected by such errors. In any case, our main interest is for high frequencies, for which the quantitative behavior shows a power-law tail for both words and lemmas. This extends for almost three orders of magnitude, with exponents *γ* very close to 2, implying the fulfillment of Zipf’s law (see Table 2).

**Table 2:** Power-law fitting results for words and lemmas, denoted respectively by subindices *w* and *l*. *V* is the number of types (vocabulary size), *n*_*m*_ is the maximum frequency of the distribution, *N*_*a*_ is the number of types in the power-law tail, i.e., with *n* ≥ *a*, *a* is the minimum value for which the power-law fit holds, and *γ* and *σ* are the power-law exponent and its standard deviation, respectively. 2*σ*_*d*_, the double of the standard deviation *σ*_*d*_ is also given. *σ*_*d*_ is the standard deviation of *γ*_*l*_−*γ*_*w*_ assuming independence, which is $\sigma_{d}=\sqrt{\sigma_{w}^{2}+\sigma_{l}^{2}}$. The last column provides ℓ_1_, the number of lemmas associated to only one word form. Notice that the lemma exponent is very close to the one found in Ref. [29] for the tail of a double power-law fitting, except for *Moby-Dick* and *Ulysses*.

Title	*V*_*w*_	*n*_*mw*_	*N*_*a*_*w*__	*a*_*w*_	*γ*_*w*_ ± *σ*_*w*_	*V*_*l*_	*n*_*ml*_	*N*_*a*_*l*__	*a*_*l*_	*γ*_*l*_ ± *σ*_*l*_	2*σ*_*d*_	ℓ_1_
Clarissa	20492	38632	1514	51	1.83±0.02	9041	41679	838	101	1.83±0.03	0.07	5750
Moby-Dick	18516	14438	2658	8	1.97±0.02	9141	14438	1548	13	1.90±0.02	0.06	6157
Ulysses	29450	14934	4377	6	1.95±0.01	12469	14934	1024	26	1.97±0.03	0.07	8670
Don Quijote	21180	20704	939	40	1.93±0.03	7432	31521	936	32	1.83±0.03	0.08	3812
La Regenta	21871	19596	1196	26	2.01±0.03	9900	32300	993	32	2.00±0.03	0.08	5308
Artamène	25161	88490	936	200	1.86±0.03	5008	119016	641	200	1.79±0.03	0.08	2178
Bragelonne	25775	26848	3173	16	1.84±0.02	10744	45577	1382	40	1.84±0.02	0.06	5391
Seitsemän	22035	4247	22035	1	2.13±0.01	7658	4247	474	26	2.13±0.05	0.10	4246
Kevät ja	25071	5042	8660	2	2.05±0.01	8898	6886	699	20	1.96±0.04	0.07	5060
Vanhempieni	35931	5254	6523	3	2.09±0.01	13510	7526	571	32	2.05±0.04	0.09	7837

The rest of books analyzed show a similar qualitative behavior, as shown for 4 of them in Fig 1(b). In all cases Zipf’s law holds, both for words and for lemmas. The power-law tail exponents *γ* range from 1.83 to 2.13, see Table 2, covering from 2 and a half to 3 and a half orders of magnitude of the type frequency (except for lemmas in *Seitsemän veljestä*, with roughly only 2 orders of magnitude). For the second power-law regime reported in Ref. [26] for the high-rank domain of lemmas (i.e., low lemma frequencies), we only find it for the smallest frequencies (i.e., between *n* = 1 and a maximum *n*) in two Finnish novels, *Kevät ja takatalvi* and *Vanhempieni romaani* with exponents *γ* = 1.715 and 1.77±0.008, respectively. These values of *γ* yield *α* = 1.40 and 1.30 (recall Eq (3)), which one can compare to the value obtained in Ref. [26] for a Polish novel (1.52). However, for the rest of distributions of lemma frequency, a discrete power law starting in *n* = 1 is rejected no matter the value of the maximum *n* considered. This is not incompatible with the results of Ref. [29], as a different fit and a different testing procedure was used there. Note that the Finnish novels yield the poorest statistics (as their text lengths are the smallest), so this second power-law regime seems to be significant only for short enough texts.

### The consistency of the exponents between word forms and lemmas

**Fig 2:**
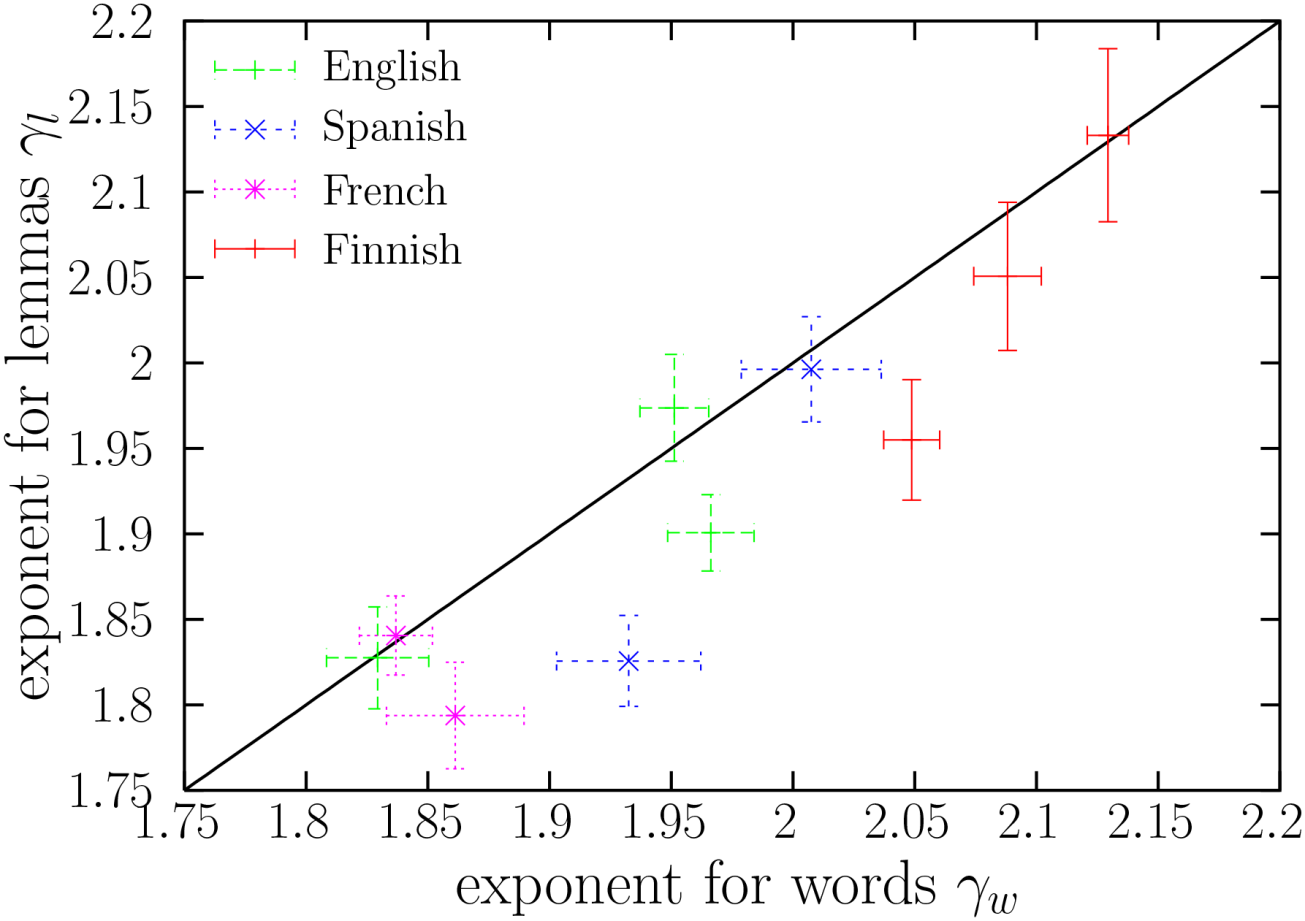
*γ*_*l*_ (the exponent of the frequency distribution of lemmas) versus *γ*_*w*_ (the exponent of the frequency distribution of word forms). As a guide to the eye, the line *γ*_*l*_ = *γ*_*w*_ is also shown (solid line). Error bars indicate one standard deviation.

In order to proceed with the comparison between the exponents of the frequency distributions of words (*w*) and lemmas (*l*), let us denote them as *γ*_*w*_ and *γ*_*l*_, respectively. Those values are compared in Fig 2. Coming back to the example of *La Regenta*, it is remarkable that the two exponents do not show a noticeable difference (as it is apparent in Fig 1(a)), with values *γ*_*w*_ = 2.01 ± 0.03 and *γ*_*l*_ = 2.00 ± 0.03. Out of the remaining 9 texts, 4 of them give pairs of word-lemma exponents with a difference of 0.02 or smaller. This is within the error bars of the exponents, represented by the standard deviations *σ*_*w*_ and *σ*_*l*_ of the maximum likelihood estimations of the exponents; more precisely, ∣*γ*_*w*_ − *γ*_*l*_∣ < *σ*_*l*_, as can be seen in Table 2. For the other 5 texts, the two exponents are always in the range of overlap of two standard deviations, i.e., ∣*γ*_*w*_ − *γ*_*l*_∣ < 2(*σ*_*w*_ + *σ*_*l*_).

However, we should be cautious in drawing conclusions from these data. If, for a fixed book, *γ*_*w*_ and *γ*_*l*_ were independent variables, the standard deviation of their difference would be $\sigma_{d}=\sqrt{\sigma_{w}^{2}+\sigma_{l}^{2}}$, according to elementary probability theory ([40], Chapter 3); however, independence cannot be ensured and we have $\sigma_{d}=\sqrt{\sigma_{w}^{2}+\sigma_{l}^{2}-2\text{cov}(\gamma_{w},\gamma_{l})}$, where cov(*γ*_*w*_, *γ*_*l*_) is the covariance of both variables, for a fixed book (this covariance is different from the covariance implicit in the Pearson correlation introduced below, which refers to all texts). Although the maximum likelihood method provides an estimation for the standard deviations of the exponents (for a fixed text) [23, 38], we cannot compute the covariance of the word and lemma exponents (for the total size of each text), and therefore we do not know the uncertainty in the difference between them. This is is due to the fact that we only have one sample for each book to calculate *γ*_*w*_ and *γ*_*l*_. If we could assume independence, we would obtain that already three books yield results outside the 95% confidence interval of the exponent difference (given by 2*σ*_*d*_), see Table 2. This could be modified somewhat by the Bonferroni and Šidák corrections for multiple testing [41, 42]. Nevertheless, we expect a non-zero covariance between *γ*_*w*_ and *γ*_*l*_, as the samples representing words and lemmas have some overlap (for instance, some word tokens remain the same after lemmatization), and therefore the standard deviation *σ*_*d*_ should be smaller than in the independent case, which leads to larger significant differences than what the independence assumption yields. Conversely, the standard deviations *σ*_*w*_ and *σ*_*l*_ of the maximum likelihood exponents are obtained assuming that *a*_*w*_ and *a*_*l*_ are fixed parameters, but they are not, and then the total uncertainties of the exponents are expected to be larger than the reported standard deviations; nevertheless, this is difficult to quantify. Thus, the standard deviations we provide for the exponents have to be interpreted as some indication of their uncertainty but not as the full uncertainty, which could be larger. We conclude that we cannot establish an absolute invariance of the value of the Zipf exponent under lemmatization.

Instead of comparing the word and lemma exponents book by book, using the uncertainty for each exponent, we can also deal with the whole ensemble of exponents, ignoring the individual uncertainties. We consider first a Student’s *t*–test for paired samples to analyze the differences between pairs of exponents. This test, although valid for dependent normally distributed data (and the estimations of the exponents are normally distributed), assumes that the standard deviations *σ*_*w*_ and *σ*_*l*_ are the same for all books, which is not the case, see Table 2. So, as a first approximation we apply the test and interpret its results with care. The *t*–statistics gives *t* = 2.466 (*p*-value = 0.036), leading to the rejection of the hypothesis that there are no significant differences between the exponents. These results do not look like very surprising upon visual inspection of Fig 2: Most points (*γ*_*w*_, *γ*_*l*_) lie below the diagonal, suggesting a tendency for *γ*_*l*_ to have a lower value than *γ*_*w*_. But we can go one step further with this test and consider the existence of one outlier, removing from the data the book with the largest difference between their exponents. In this case one needs to avoid introducing any bias in the calculation of the *p*-value. For this purpose, we simulate the *t*–Student distribution by summing rescaled normal variables in the usual way (see Materials and Methods), and remove (in the same way as for empirical data) the largest value of the variables. This yields *t* = 2.053 and *p* = 0.075, which suggests that the values of the exponents are not significantly different, except for one outlier. However, as we have mentioned, this test cannot be conclusive and other tests are necessary.

We realize that *γ*_*w*_ and *γ*_*l*_ are clearly dependent variables (when considering all books). Their Pearson correlation, a measure of linear correlation, is *ρ* = 0.913 (the sample size is 𝓝 = 10 and *p* = 0.0003 is the *p*-value of a two-sided test with null hypothesis *ρ* = 0). Note that this correlation is different to the one given above by cov(*γ*_*w*_, *γ*_*l*_), which referred to a fixed book. Given this, we formulate three hypotheses about the relationship between the exponents. The first hypothesis is that *γ*_*w*_ and *γ*_*l*_ are identically distributed for a given text (but not necessarily for different texts, different authors, or different languages). The second hypothesis is that *γ*_*w*_ is centered around *γ*_*l*_, i.e., the conditional expectation of *γ*_*w*_ given *γ*_*l*_ is *E*[*γ*_*w*_∣*γ*_*l*_] = *γ*_*l*_. This means that a reasonable prediction on the value of *γ*_*w*_ can be attained from the knowledge of the value of *γ*_*l*_. The third hypothesis is the symmetric of the second, namely that *γ*_*l*_ is centered around *γ*_*w*_, i.e., the conditional expectation of *γ*_*l*_ given *γ*_*w*_ is *E*[*γ*_*l*_∣*γ*_*w*_] = *γ*_*w*_. The second and third hypotheses are supported by the strong Pearson correlation between *γ*_*w*_ and *γ*_*l*_, but these two hypotheses are not equivalent [43].

We define ${\bar{\gamma }}_{w}$ and ${\bar{\gamma }}_{l}$ as the average values of *γ*_*w*_ and *γ*_*l*_, respectively, in our sample of ten literary texts. The first hypothesis means that given a certain text, *γ*_*w*_ and *γ*_*l*_ are interchangeable. If *γ*_*w*_ and *γ*_*l*_ are identically distributed for a certain text, then the absolute value of the difference between the means $|{\bar{\gamma }}_{w}-{\bar{\gamma }}_{l}|$ should not differ significantly from analogous values obtained by chance, i.e., flipping a fair coin to decide if *γ*_*w*_ and *γ*_*l*_ remain the same or are swapped within a book. As there are ten literary texts, there are 2^10^ possible configurations. Thus, one can compute numerically the *p*-value as the proportion of these configurations where $|{\bar{\gamma }}_{w}-{\bar{\gamma }}_{l}|$ equals or exceeds the original value. This coin-flipping test is in the same spirit as Fisher’s permutational test ([44], pp. 407–416), with the difference that we perform the permutations of the values of the exponents only inside every text. The application of this test reveals that $|{\bar{\gamma }}_{w}-{\bar{\gamma }}_{l}|=0.035$, which is a significantly large difference (with a *p*-value = 0.04). Therefore, we conclude that the first hypothesis does not stand, and therefore *γ*_*w*_ and *γ*_*l*_ are not identically distributed within books. This seems consistent with the fact that most points (*γ*_*w*_, *γ*_*l*_) lay below the diagonal, see Fig 2. However, the elimination of one outlier (the text with the largest difference) leads to *p* = 0.08, which makes the difference non-significant for the remaining texts.

The second hypothesis is equivalent to *E*[*γ*_*w*_/*γ*_*l*_∣*γ*_*l*_] = 1 and therefore this hypothesis is indeed that the ratio *γ*_*w*_/*γ*_*l*_ is mean independent of *γ*_*l*_ (the definition of mean independence in this case is *E*[*γ*_*w*_/*γ*_*l*_∣*γ*_*l*_] = constant = *E*[*γ*_*w*_/*γ*_*l*_], ([43], pp. 67)). Similarly, the third hypothesis is equivalent to *E*[*γ*_*l*_/*γ*_*w*_∣*γ*_*w*_] = 1 and therefore this hypothesis is indeed that *γ*_*l*_/*γ*_*w*_ is mean independent of *γ*_*w*_. Mean independence can be rejected by means of a correlation test as mean independence needs uncorrelation (see Ref. [45], pp. 60 or Ref. [43], pp. 67). A significant correlation between *γ*_*w*_/*γ*_*l*_ and *γ*_*l*_ would reject the second hypothesis while a significant correlation between *γ*_*l*_/*γ*_*w*_ and *γ*_*w*_ would reject the third hypothesis. Table 3 indicates that neither the Pearson nor the Spearman correlations are significant (see Materials and Methods), and therefore these correlation tests are not able to reject the second and the third hypotheses. Further support for the second and third hypotheses comes from linear regression. The second hypothesis states that *E*[*γ*_*w*_∣*γ*_*l*_] = *c*_1_ *γ*_*l*_+*c*_2_ with *c*_1_ = 1 and *c*_2_ = 0 while the third hypothesis states that *E*[*γ*_*l*_∣*γ*_*w*_] = *c*_3_ *γ*_*w*_+*c*_4_ with *c*_3_ = 1 and *c*_4_ = 0. Consistently, a standard linear regression and subsequent statistical tests indicate that *c*_1_, *c*_3_ ≈ 1 and *c*_2_, *c*_4_ ≈ 0 cannot be rejected (Table 4).

**Table 3:** Analysis of the association between random variables using Pearson and Spearman correlations as statistics. *ρ* is the value of the correlation statistic and *p* is the *p*-value of a two-sided test with null hypothesis *ρ* = 0, calculated through permutations of one of the variables (the results can be different if *p* is calculated from a *t*–test). The sample size is 𝓝 = 10 in all cases. Only the Spearman correlation between *a*_*w*_ and *a*_*l*_/*a*_*w*_ is significantly different from zero.

Association	Correlation test	*ρ*	*p*
*γ*_*w*_/*γ*_*l*_ and *γ*_*l*_	Pearson correlation test	−0.378	0.28
	Spearman correlation test	−0.418	0.23
*γ*_*l*_/*γ*_*w*_ and *γ*_*w*_	Pearson correlation test	−0.034	0.92
	Spearman correlation test	−0.091	0.81
*a*_*w*_/*a*_*l*_ and *a*_*l*_	Pearson correlation test	0.420	0.24
	Spearman correlation test	0.393	0.26
*a*_*l*_/*a*_*w*_ and *a*_*w*_	Pearson correlation test	−0.373	0.11
	Spearman correlation test	−0.867	0.002

**Table 4:** The fit of a linear model for the relationship between exponents (*γ*_*w*_ and *γ*_*l*_) and the relationship between cut-offs (*a*_*w*_ and *a*_*l*_). *c*_1_ and *c*_3_ stand for slopes and *c*_2_ and *c*_4_ stand for intercepts. The error bars correspond to one standard deviation. A Student’s *t*-test is applied to investigate if the slopes are significantly different from one and if the intercepts are significantly different from zero. The resulting *p*-values indicate that in all cases the slopes are compatible with being equal to one. The intercepts are compatible with zero for the exponents, but seem to be incompatible for the cut-offs.

Linear model	Parameters	Student’s *t*	*p*
*E*[*γ*_*w*_∣*γ*_*l*_] = *c*_1_ *γ*_*l*_ + *c*_2_	*c*_1_ = 0.855 ± 0.135	−1.074	0.314
	*c*_2_ = 0.315 ± 0.261	1.208	0.261
*E*[*γ*_*l*_∣*γ*_*w*_] = *c*_3_ *γ*_*w*_ + *c*_4_	*c*_3_ = 0.975 ± 0.154	−0.161	0.876
	*c*_4_ = 0.013 ± 0.303	0.044	0.966
*E*[*a*_*w*_∣*a*_*l*_] = *c*_1_ *a*_*l*_ + *c*_2_	*c*_1_ = 1.012 ± 0.103	0.115	0.911
	*c*_2_ = −17.523±7.798	−2.247	0.055
*E*[*a*_*l*_∣*a*_*w*_] = *c*_3_ *a*_*w*_ + *c*_4_	*c*_3_ = 0.912 ± 0.093	−0.945	0.372
	*c*_4_ = 20.009 ± 6.272	3.190	0.013

In any case, to perform our analysis we have not taken into account that the number of datapoints (*V*) and the power-law fitting ranges are different for words and lemmas, a fact that can increase the difference between the values of the exponents (due to the fact that the detection of deviations from power-law behavior depends on the number of datapoints available). In general, the fitting ranges are larger for words than for lemmas, due to the bending of the lemma distributions, see below. Another source of variation to take into account for the difference between the exponents is, as we have mentioned, that the lemmatization process is not exact, which can lead to type assignment errors and even to some words not being associated to any lemma (see the Materials and Methods Section for details).

Although, after the elimination of one outlier, we are not able to detect differences between the exponents, there seems to be a tendency for the lemma exponent to be a bit smaller than the word exponent, as can be seen in Fig 2. This can be an artifact of the fitting procedure, which can yield fitting ranges that include a piece of the bending-downwards part of the distribution in the case of lemmas. The only way to avoid this would be either to have infinite data, or not to find the fitting range automatically, or to use a fitting distribution that parametrizes also the bending. As we are mostly interested in the power-law regime, we have not considered these modifications to the fits.

**Fig 3:**
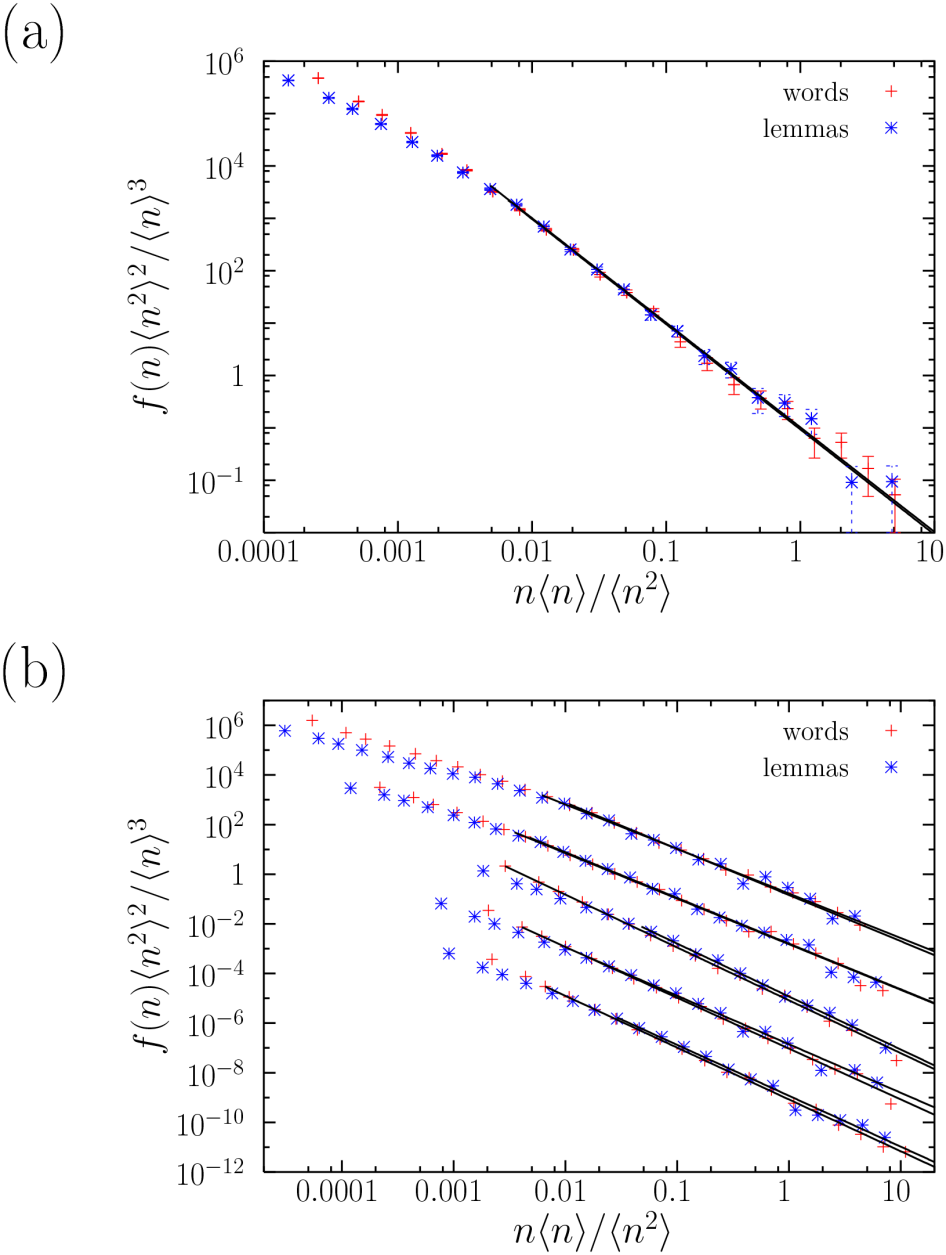
(a) Probability mass functions *f*(*n*) of the absolute frequencies *n* of words and lemmas in *La Regenta*, together with their fits, under rescaling of both axis. The collapse of the tails indicates the compatibility of both power-law exponents. (b) The same for, from top to bottom, *Artamène, Bragelonne* (both in French), *Seitsemän v., Kevät ja t*., and *Vanhempieni r*. (all three in Finnish). The rescaled distributions are multiplied in addition by factors 1, 10^−2^, etc., for a clearer visualization.

A rescaling of the axes as in Refs. [36, 46] can lead to additional support for our results (see also Ref. [29]). Fig 3(a) shows the rescaling for *La Regenta*. Each axis is multiplied by a constant factor, in the form

$$\begin{array}{ccc} n & \to & n\left\langle n\right\rangle /\left\langle n^{2}\right\rangle \\ f(n) & \to & f\left(n\right){\left\langle n^{2}\right\rangle }^{2}/{\left\langle n\right\rangle }^{3}, \end{array}$$

which translates into a simple shift of the curves in a double-logarithmic plot, not affecting the shape of the distribution and therefore keeping the possible power-law dependence. The collapse of the tails of the two curves into a single one is then an alternative visual indication of the stability of the exponents. The results for the 5 texts that were not shown before are now displayed in Fig 3(b). These findings suggest that, in general, Zipf’s law fulfills a kind of invariance under lemmatization, at least approximately, although there can be exceptions for some texts.

Finally, in order to test the influence of the stream of consciousness part of *Ulysses* on the results, we have repeated the fits removing that part of the text. This yields a new text that is about 9% shorter, but more homogeneous. The Zipf exponents turn out to be *γ*_*w*_ = 1.98±0.01 for *n* ≥ 6 and *γ*_*l*_ = 2.02±0.04 for *n* ≥ 32, slightly higher than for the complete text. Nevertheless, the new *γ*_*w*_ and *γ*_*l*_ still are compatible between them (in the sense explained above for individual texts), and therefore our conclusions do not change regarding the similarity between word and lemma exponents. If we pay attention to the removed part, despite its peculiarity, the stream of consciousness prose still fulfills Zipf’s law, but with smaller exponents, *γ*_*w*_ = 1.865±0.02 for *n* ≥ 2 and *γ*_*l*_ = 1.82±0.03 for *n* ≥ 3. Both exponents are also compatible between them.

### The consistency of the lower cut-offs of frequency for word forms and lemmas

**Fig 4:**
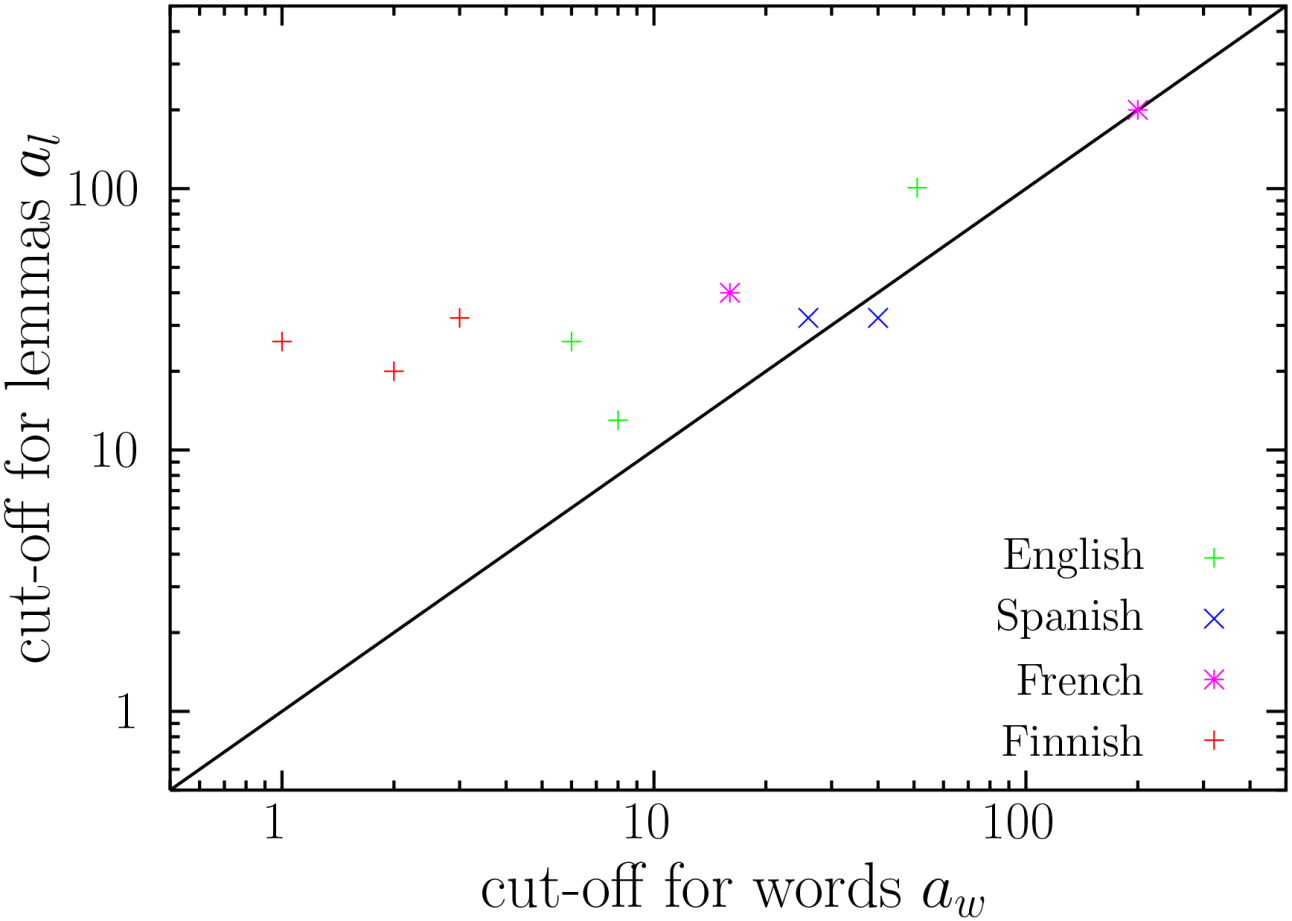
The lower cut-off for the frequency distribution of lemmas (*a*_*l*_) versus the lower cut-off for the frequency distribution of word forms (*a*_*w*_). The line *a*_*l*_ = *a*_*w*_ is also shown (solid line).

As we have done with the exponent *γ*, we define *a*_*w*_ and *a*_*l*_ as the lower cut-off of the power-law fit for the frequency distributions of words and of lemmas, respectively. Those values are compared in Fig 4. When all texts are considered, a Student *t*–test for paired samples yields the rejection of the hypothesis that there is no significant difference in the values of *a*_*w*_ and *a*_*l*_, even if the presence of one possible outlier is taken into account (*t* = −3.091 and *p* = 0.015). In fact, *a*_*w*_ and *a*_*l*_ are not independent, as their Pearson correlation is *ρ* = 0.961 (𝓝 = 10 and *p* = 0.0014 for the null hypothesis *ρ* = 0, calculated through permutations of one of the variables). These results are not very surprising upon inspection of Fig 4: Most points (*a*_*w*_, *a*_*l*_) lay above the diagonal, suggesting a tendency for *a*_*l*_ to exceed *a*_*w*_.

Like we did for the exponents, we formulate three hypotheses about the relationship between the low-frequency cut-offs. The first hypothesis is that *a*_*w*_ and *a*_*l*_ are identically distributed for a given text. The second hypothesis is that the expectation of *a*_*w*_ given *a*_*l*_ is *E*[*a*_*w*_∣*a*_*l*_] = *a*_*l*_, while the third hypothesis is that the expectation of *a*_*l*_ given *a*_*w*_ is *E*[*a*_*l*_∣*a*_*w*_] = *a*_*w*_. The second and third hypotheses are supported by the strong Pearson correlation between *a*_*w*_ and *a*_*l*_ just mentioned. We define ${\bar{a}}_{w}$ and ${\bar{a}}_{l}$ as the mean value of *a*_*w*_ and *a*_*l*_, respectively, in our sample of ten texts. The coin flipping test reveals that $|{\bar{a}}_{w}-{\bar{a}}_{l}|=16.9$ is significantly high (*p*-value = 0.01). Therefore, the first hypothesis does not stand, not even after the exclusion of one outlier (which leads to *p* = 0.03).

The second hypothesis is indeed that *a*_*w*_/*a*_*l*_ is mean independent of *a*_*l*_ while the third hypothesis is that *a*_*l*_/*a*_*w*_ is mean independent of *a*_*w*_. Table 3 indicates that neither a Pearson nor a Spearman correlation test are able to reject the second hypothesis. In contrast, a Pearson correlation test fails to reject the third hypothesis but the Spearman correlation test does reject it. This should not be interpreted as an contradiction between Pearson and Spearman tests but as an indication that the relationship between *a*_*l*_ and *a*_*w*_ is non-linear, as suggested by Fig 4. As a typical correlation test is conservative because it only checks a necessary condition for mean dependence [47], a further test is required. The second hypothesis states that *E*[*a*_*w*_∣*a*_*l*_] = *c*_1_ *a*_*l*_ + *c*_2_ with *c*_1_ = 1 and *c*_2_ = 0 while the third hypothesis states that *E*[*a*_*l*_∣*a*_*w*_] = *c*_3_ *a*_*w*_ + *c*_4_ with *c*_3_ = 1 and *c*_4_ = 0. A standard linear regression indicates that *c*_1_, *c*_3_ ≈ 1 but *c*_2_ ≈ 0 is in the limit of rejection, whereas *c*_4_ ≈ 0 fails (Table 4). Therefore, this suggests that the cut-offs do not follow hypothesis 3. Note that the significance of the values *c*_2_ < 0 and *c*_4_ > 0 implies that, in general, *a*_*l*_ is significantly larger than *a*_*w*_. This is consistent with Fig 4.

## Discussion

We have shown that Zipf’s law is fulfilled in long literary texts for several orders of magnitude in word and lemma frequency. The exponent of lemmas and the exponent of word forms are positively correlated. Similarly, the low-frequency cut-offs of lemmas and that of word forms are positively correlated. However, the exponent is more stable than the cut-off under the lemmatization transformation. While the exponent of lemmas is apparently centered around that of word forms and vice versa, the equivalent relationships are not supported for the cut-offs. However, we cannot exclude the possibility that the exponents of lemmas are indeed not centered around those of word forms. Some suspicious evidence comes from Fig 2, where it can clearly be seen that *γ*_*l*_ ≤ *γ*_*w*_ in most cases. The tendency to satisfy this inequality is supported by the slight increase of the exponent *α* when moving from words to lemmas that has been reported in previous research [26, 27] and that we have reviewed in the Introduction. Although Refs. [26, 27] employed methods that differ substantially from ours, Eq (3) allows one to interpret, with some approximation, the increase from *α*_*w*_ to *α*_*l*_ of Refs. [26, 27] as the drop from *γ*_*w*_ to *γ*_*l*_ we have found in most cases. The apparent stability of the exponent of Zipf’s law could be a type II error caused by the current size of our sample of long single-author texts. Furthermore, the apparently constant relationship between *γ*_*l*_/*γ*_*w*_ and *γ*_*w*_ (or between *γ*_*w*_/*γ*_*l*_ and *γ*_*l*_) may hide a non-monotonic dependence, which the correlation tests above are blind to (our correlation tests are biased towards the detection of monotonic dependences). In spite of these limitations, one conclusion is clear: Exponents are more stable than cut-offs.

**Fig 5:**
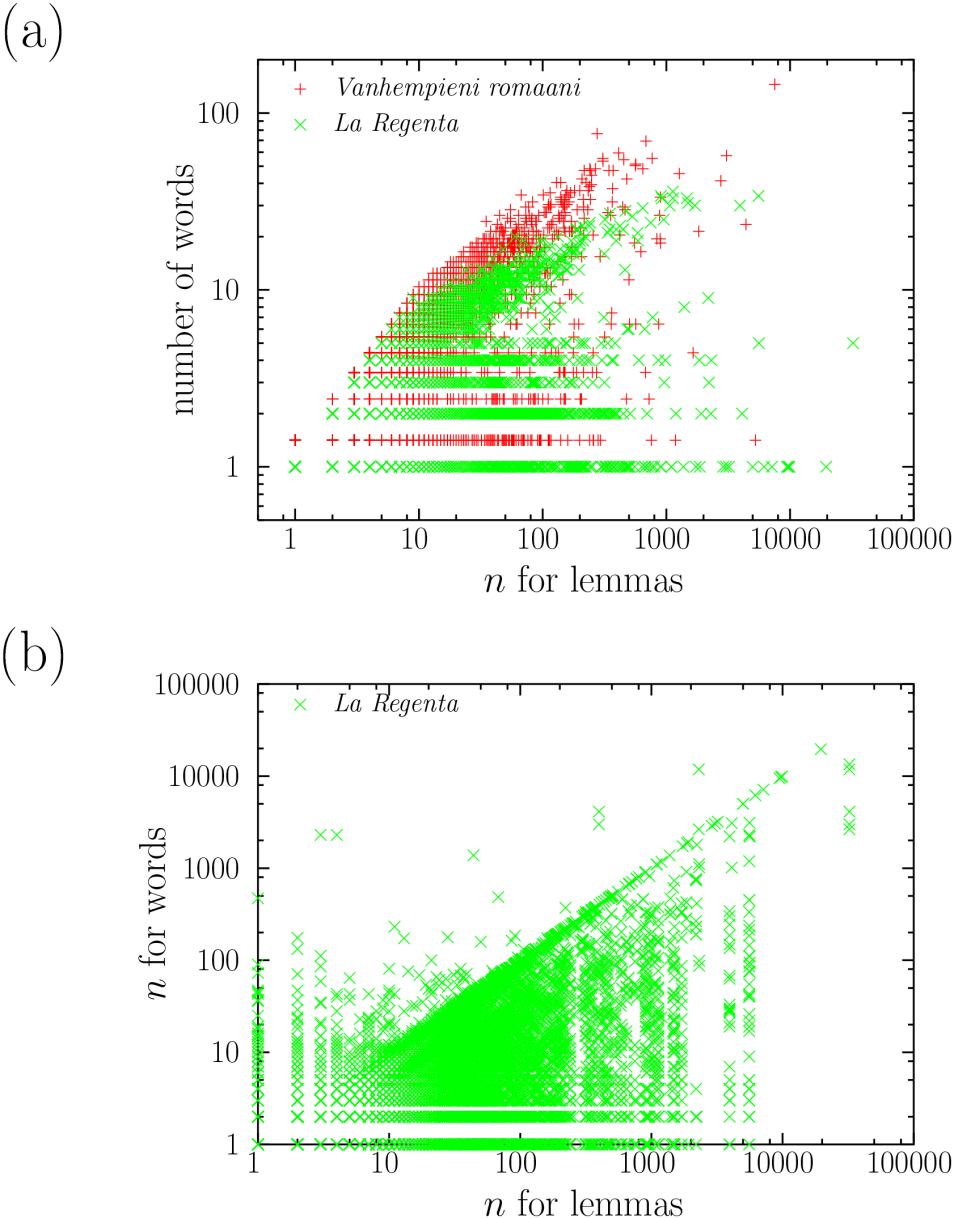
(a) Number of words per lemma as a function of lemma absolute frequency *n*_*l*_ in *Vanhempieni romaani* (in Finnish) and in *La Regenta*. The figures for the former have been slightly shifted up for clarity sake. (b) Frequency of words *n*_*w*_ as a function of the frequency of their lemmas *n*_*l*_ in *La Regenta*.

The similarity between the exponents of words and lemmas would be trivial if the lemmatization process affected only a few words, or if these words were those with the smallest values of the frequency (where the two distributions are more different). However, Fig 5(a) displays the number of words that corresponds to each lemma for *La Regenta* and for *Vanhempieni romaani* (in Finnish), showing that the effect of lemmatization is rather important [33]. Lemmatization affects all frequency scales, and, in some cases, almost 50 words are assigned to the same lemma in Spanish (verb paradigms), and more than 100 in Finnish (lemma *olla*). All texts in Spanish, French, and Finnish yield very similar plots; texts in English lead to flatter plots, because lemmatization is not such a big transformation there due to the morphological characteristics of English. Fig 5(b) shows the same effect in a different way, depicting the frequency of each word as a function of the frequency of its corresponding lemma. The presence of data above the diagonal is due to the fact that some words can be associated to more than one lemma, and then the sum of the frequencies of the words corresponding to one lemma is not the frequency of the lemma; this is the case in English of the word *found*, which can correspond to two lemmas, *(to) found* or *(to) find*.

**Fig 6:**
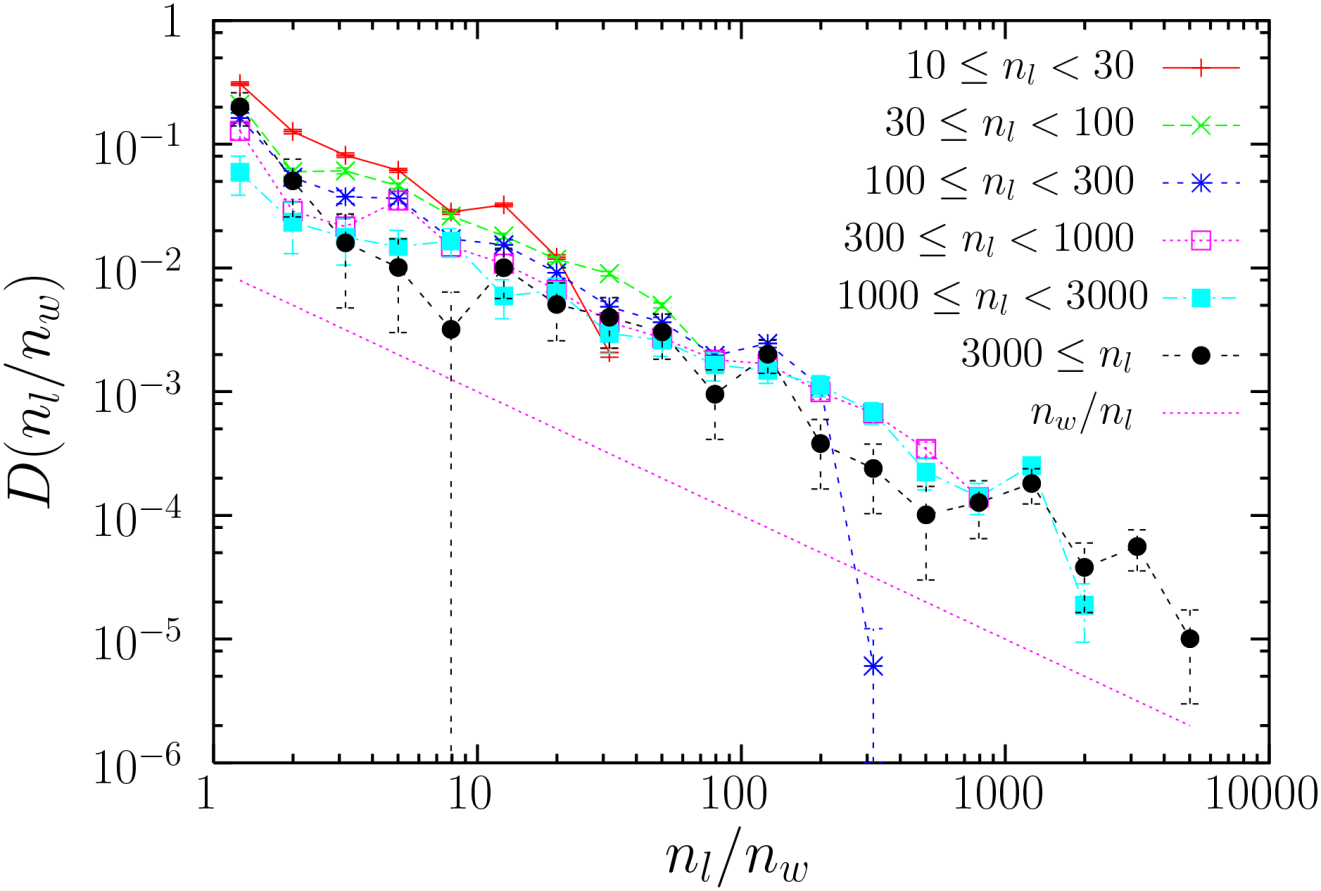
Probability density *D*(*n*_*l*_/*n*_*w*_) of the frequency ratio for lemmas and words, *n*_*l*_/*n*_*w*_, in *La Regenta*. Values of *n*_*l*_ smaller than *n*_*w*_ are disregarded, as they arise from words associated to more than one lemma. Bending for the largest *n*_*l*_/*n*_*w*_ is expected as the maximum of the ratio is given by *n*_*l*_, which is not constant for each distribution but has a variation of half an order of magnitude (see plot legend).

Finally, a complementary view is provided in Fig 6, which shows the distribution of the ratio of frequencies *n*_*l*_/*n*_*w*_ for the words that correspond to a given lemma (the subindices refer to lemmas and words, respectively). In all cases this ratio is broadly distributed, resembling a power law, although the statistics is too poor to draw more solid conclusions. As an indication, we plot in the figure a power law with exponent around 1, which is a good visual guide for texts in Spanish and French. In Finnish, the distribution becomes broader, being closer to a power law with exponent 0.5, whereas in English the decay is faster, around an exponent 1.5 (not shown). In any case, the relation between the frequency of words and the frequency of their lemmas seems to lack a characteristic scale. The simplest case in which there is only one word per lemma (and then their frequencies are the same, *n*_*l*_/*n*_*w*_ = 1) is quantified in the last column of Table 2.

A challenge for future research is to illuminate the approximated invariance in the word-lemma transformation. A simplistic approach is offered by MacArthur’s broken-stick model for species abundances [48]. Assume that each lemma, with frequency *n*, only “breaks” into two different words, with frequencies in the text given by *m* and *n* − *m*. If *m* is distributed uniformly between 0 and *n*, and the distribution of lemma frequencies is a power law, then, the distribution of word frequencies *m* turns out to be also a power law, with the same exponent (see the supplementary information of Ref. [49]). However, there is a long way from this oversimplification to reality. We have learned in Fig 5(a) that the number of words a lemma can yield varies a lot, from a few words for nominal lemmas to many words for verb lemmas in Spanish or French. More realistic models from an evolutionary perspective certainly appear as avenues for future work.

## Conclusions

We have studied the robustness of Zipf’s law under lemmatization for single-author written texts. For this purpose it is crucial to unambiguously determine the power-law exponent *γ* of the frequency distribution of types, and the range of validity of Zipf’s law, given by the low-frequency cut-off *a*, both for unlemmatized texts (consisting of word forms) and for lemmatized texts (transformed into sequences of lemmas). We find that word and lemma distributions are somewhat different, but the exponents of Zipf’s law in both cases remain close to each other, for most of the texts, especially when compared to cut-offs. Nevertheless, the set of values of *γ* suggests a slight bias for the exponents of lemmas to decrease with respect to that of words. In contrast to the exponents, the cut-offs we find are not stable at all under the lemmatization transformation, but are significantly increased, which in turn implies a decrease in the range of validity of Zipf’s law. Random breaking of lemmas into words might explain the relative stability of the power-law distribution under the lemma-word transformation, but cannot account for the wider validity of Zipf’s law for words.

As Zipf’s law is a paradigm that goes beyond linguistics, having been found in the distribution of number of city inhabitants [50] or in the size of companies [51] (among many other systems in which “tokens” merge to constitute “types” [23]), our results could have a much broader applicability. In many of these cases, the aggregation of tokens to form types can be done in different ways, or types can be merged themselves to constitute “supertypes”, in a coarse-grained process akin both to lemmatization and to a transformation of the renormalization group [52]. This is what was attempted in Refs. [53, 54], where the spatial extent of elementary patches was added to define what was called there a natural city. Extrapolating our results, we could expect that Zipf’s exponent for city areas would not be very much affected by this process; in that case, the changes in Zipf’s *α* exponent found in Ref. [53] indicate that further study is necessary to elucidate whether the differences arise from the data (and so are due to differences in the underlying phenomenon) or from the data manipulation, e.g. the fitting method. In general, investigating the commonalities and differences between different systems displaying Zipf’s law is an area that should be actively addressed in the near future.

## Materials and Methods

### Corpus selection

First, we selected languages we have some command of (for data and error analysis purposes) and there are freely available lemmatization tools for [55, 56]. The exception is Finnish, which we included because it is a morphologically rich language that could shed light on the impact of lemmatization processes in Zipf’s law. We were interested in finding very long texts by single authors, and with that purpose we searched for the longest literary texts ever written. Of those novels published by mainstreaming publishers, *Artamène* is ranked as the longest, in any language, and *Clarissa* as the longest in English [57]. *Don Quijote*, consistently considered the best literary piece ever written in Spanish, is also of considerable length. The list was completed based on the availability of an electronic version of the novels in the *Project Gutenberg* [58]. Note that *Artamène* was not found in the *Gutenberg Project* but in a different source [59]. We were not able to find novels in Finnish of comparable length to those in the other languages and in this case they are much shorter, see Table 1.

### Lemmatization

To carry out the comparison between word forms and lemmas, texts must be lemmatized. A manual lemmatization would have exceeded the possibilities of this project, so we employed natural language processing tools: *FreeLing* [55] for Spanish and English, *TreeTagger* [56] for French, and *Connexor*’s tools [60] for Finnish.

The tools carry out the following steps:

1. Tokenization: Segmentation of the texts into sentences and sentences into words, symbols, and punctuation marks (tokens).
2. Morphological analysis: Assignment of one or more lemmas and morphological information (a part-of-speech tag) to each token. For instance, *houses* in English can correspond to the plural form of the noun *house* or the third person singular, present tense form of the verb *to house*. At this stage, both are assigned whenever the word form *houses* is encountered.
3. Morphological disambiguation: An automatic tagger assigns the single most probable lemma and tag to each word form, depending on the context. For instance, in *The houses were expensive* the tagger would assign the nominal lemma and tag to *houses*, while in *She usually houses him*, the verb lemma and tag would be preferred. We note that as in both cases the lemma is the same, both occurrences would count in the statistics of the *house* lemma.

As all these steps are automatic, some errors are introduced at each step. However, the accuracy of the tools is quite high (e.g., around 95–97% at the token level for morphological disambiguation), such that a quantitative analysis based on the results of the automatic process can be carried out. Also note that step 2 is based on a pre-existing dictionary (of words, not of lemmas, also called a lexicon): only the words that are in the dictionary are assigned a reliable set of morphological tags and lemmas. Although most tools use heuristics to assign tag and/or lemma information to words that are not in the dictionary, the results shown in this paper are obtained by counting only tokens of lemmas for which the corresponding word types are found in the dictionary, so as to minimize the amount of error introduced by the automatic processing. This comes at the expense of losing some data. However, the dictionaries have quite a good coverage of the vocabulary, particularly at the token level, but also at the type level (see Table 5). The exceptions are *Ulysses*, because of the stream of consciousness prose, which uses many non-standard word forms, and *Artamène*, because 17th century French contains many word forms that a dictionary of modern French does not include.

**Table 5:** Coverage of the vocabulary by the dictionary in each language, both at the word-type and at the token level. The average for all texts is also included. Remember that we distinguish between a word *type* (corresponding to its orthographic form) and its *tokens* (actual occurrences in text).

Title	Tokens	Types
Clarissa	96.9%	68.0%
Moby-Dick	94.7%	70.8%
Ulysses	90.4%	58.6%
Don Quijote	97.0%	81.3%
La Regenta	97.9%	89.5%
Artamène	83.6%	43.6%
Bragelonne	97.5%	89.8%
Seitsemän v.	95.4%	89.8%
Kevät ja t.	98.3%	96.2%
Vanhempieni r.	98.5%	96.5%
average	95.0%	78.4%

Note that the tools we have used do not only provide lemmatization, but also morphological analysis. That means that words are associated with a lemma (*houses*: *house*) and a morphological tag (*houses*: NNS, for *common noun in plural form*, or VBZ, for *verb in present tense, third person singular*). Tags express the main part of speech (POS; for *houses*, in this case, *noun* vs. *verb*) plus additional morphological information such as number, gender, tense, etc. That means that instead of reducing our vocabulary tokens to their lemmas, we could have chosen to reduce them to their lemma plus tag information (lemma-tag, *house-NNS* vs. *house-VBZ*), or to their lemma plus POS information (lemma-POS: *house-N* vs. *house-V*). Table 6 shows that, from all these reductions, pure lemmatization (*houses*: *house*) is the most aggressive one, while still being linguistically motivated, as it reduces the size of vocabulary *V* a factor which is between 2 (for *Moby-Dick*) and 5 (for *Artamène*). Therefore, in this paper we focus on comparing word tokens with lemmas. A further reduction in the lemmatization transformation is provided by our requirement, explained in the previous paragraph, that the corresponding word is included in the dictionary of the lemmatization software. If this restriction is eliminated, the results are very similar, as the restriction mainly operates at the smallest frequencies (let us say, *n* ≤ 10 or 20), whereas the power law fit takes place for larger frequencies (see Table 2). Alternatively to lemmatization, there is a different transformation that, instead of aggregating words into lemma-POS or lemmas, segregates words into what we may call word-lemma-tag. Table 6 shows that this transformation is not very significant, in terms of changes in the size of the vocabulary.

**Table 6:** Size of vocabulary *V* (i.e., number of types) when texts are decomposed in different sorts of types, being these: word-lemma-tag (w-l-t), plain words, lemma-POS (l-pos), lemma-POS of words in the dictionary (l-pos dic), lemmas, and lemmas of words in the dictionary (lemma dic). The latter provide the most radical transformation, as it yields the largest reduction in resulting vocabulary.

	w-l-t	word	l-pos	l-pos dic	lemma	lemma dic
Clarissa	23624	20492	17058	10315	15356	9041
Moby-Dick	20777	18516	15774	10426	14226	9141
Ulysses	32952	29450	26412	14136	24089	12469
Don Quijote	23359	21180	11872	7906	11128	7432
La Regenta	24053	21871	12509	10500	11768	9900
Artamène	31574	25161	7605	5349	7177	5008
Bragelonne	28803	25775	12994	11342	12127	10744
Seitsemän	22851	22035	9749	7788	9607	7658
Kevät ja	26087	25071	9897	9054	9733	8898
Vanhempieni	37247	35931	14751	13678	14566	13510

### Statistical procedures

We now explain the different statistical tools used in the paper. We begin with the procedure to find parameter values that describe the distributions of frequencies, that is, the power-law exponent *γ* and the low-frequency cut-off *a*. As we have already mentioned, the method we adopt is based on the one by Clauset et al. [23], but it incorporates important modifications that have been shown to yield a better performance in the continuous case [36, 37]. The algorithm we use is the one described in Ref. [39].

The key issue when fitting power laws is to determine the optimum value *a* of the variable for which the power-law fit holds. The method starts by selecting arbitrary values of *a*, and for each value of *a* the maximum likelihood estimation of the exponent is obtained. In the discrete case one has to maximize the likelihood function numerically, where the normalization factor is obtained from the Hurwitz zeta function. The goodness of the fit needs to be evaluated independently. For this, the method uses the Kolmogorov-Smirnov test, and the *p*-value of the fit is obtained from Monte Carlo simulations of the fitted distribution. The simulated data need to undergo the same procedure as the original empirical data in order to avoid biases in the fit (which would lead to inflated *p*-values). In this way, for each value of *a* we obtain a fit and a quantification of the goodness of the fit given by its *p*-value. The chosen value of *a* is the smallest one (which gives the largest power-law range), provided that its *p*-value is large enough. This has an associated estimated maximum likelihood exponent, which is the final result for exponent. Its standard deviation (for the quantification of its uncertainty) is obtained, for fixed *a*, from the standard deviation of the values obtained in the Monte Carlo simulations.

The complete algorithm is implemented here with the following specifications. The minimum frequency *a* is sampled with a resolution of 10 points per order of magnitude, in geometric progression to yield a constant separation of *a*–values in logarithmic scale. The procedure is simple: A given value for *a* is obtained by multiplying its previous value by $\sqrt[{10}]{10}\approx 1.26$, with the initial value of *a* being 1, and in this sense the relative error in *a* can be considered to be of the order of $100(\sqrt[{10}]{10}-1)\approx 26\%$; the values of *a* produced that are not integers are rounded to the next integer *a posteriori* to become true parameters. The goodness of fit is evaluated with 1000 Monte Carlo simulations; and a *p*-value is considered to be large enough if it exceeds 0.20.

Now we review the methods used to investigate the similarity between words and lemmas from the perspective of the parameters of the frequency distribution. Student’s *t*–test for paired samples makes use of the differences between the values of the parameters of each text (either exponents or cut-offs, word minus lemma) and rescales the mean of the differences by dividing it by the (unbiased) standard deviation of the differences and by multiplying by $\sqrt{𝓝}$ (with 𝓝 the number of data, 10 books in our case). This yields the *t* statistic, which, if the differences are normally distributed with the same standard deviation and zero mean, follows a *t*–Student distribution with 𝓝 − 1 degrees of freedom. Simulations of 𝓝 independent normally distributed variables with zero mean and the same standard deviation mimic the distribution of the differences under the null hypothesis and lead to the *t*–Student distribution, which allows the calculation of the *p*–value. This simulation method allows for the systematic treatment of outliers, as mentioned in the main text (if one outlier is removed, then, obviously, 𝓝 = 9 in the calculation of the value of *t*).

Correlations between parameters are calculated using either the Pearson correlation coefficient or the Spearman correlation coefficient. While the Pearson coefficient is a measure of the strength of the linear association, the Spearman correlation coefficient is able to detect non-linear dependences [44, 61]. The former is defined as the covariance divided by the product of the standard deviations; the latter is defined in the same way but replacing the values of each variable by their ranks (one, two, etc.); both are represented by *ρ*. In order to test the null hypothesis *ρ* = 0 we perform a reshuffling of one of the variables and calculate the resulting *ρ*. The *p*-value is just the fraction of values of *ρ* for the reshuffled data with absolute value larger or equal than the absolute value of *ρ* for the original data (a two-sided test).

We could have also used a correlation ratio test [47], a test based on the correlation ratio, another correlation statistic [62]. That test provides a way of testing for mean independence that is *a priori* more powerful than a standard correlation test (a Pearson correlation test is a conservative test of mean dependence [47]). However, our dataset exhibits a high diversity of values (Table 2), which is known to lead to type II errors with that statistic [47].
